# Teaching with digital technology

**DOI:** 10.1007/s11858-020-01196-0

**Published:** 2020-10-23

**Authors:** Alison Clark-Wilson, Ornella Robutti, Mike Thomas

**Affiliations:** 1 UCL Institute of Education, University College London, London, UK; 2 Università di Torino, Torino, Italy; 3 Auckland University, Auckland, New Zealand

**Keywords:** Tools, Resources, Technology, Teachers, Teaching, Mathematics, Dynamic, Instrumental approach, Handheld technology, Communities of teachers, Collaboration

## Abstract

In this survey paper, we describe the state of the field of research on teaching mathematics with technology with an emphasis on the secondary school phase. We synthesize themes, questions, results and perspectives emphasized in the articles that appear in this issue alongside the relevant foundations of these ideas within the key journal articles, handbooks and conference papers. Our aim is to give an overview of the field that provides opportunities for readers to gain deeper insights into theoretical, methodological, practical and societal challenges that concern teaching mathematics with technology in its broadest sense. Although this collection of articles was developed prior to the global coronavirus pandemic, we have taken the opportunity to survey the contributing authors to provide some country perspectives on the impact the pandemic has had on mathematics teaching with technology in the period January–July 2020. We conclude the survey paper by identifying some areas for future research in this increasingly relevant topic.

## Introduction

When computers first appeared in school mathematics classes in the 1970s the emphasis was, rightly, on how they might be used to improve student learning. Early surveys, such as Schoenfeld (1988), show a variety of ways that this was attempted, ranging from drill and practice and programming to the design and use of software programs employing dynamic representations and simulations that addressed conceptual learning (Kaput, 1987). In more recent years there has been a subtle shift of emphasis on how to improve learning. This has been informed by research, such as the statistical meta-analysis of Hattie (2003), which has found clear evidence that of all the factors influencing student activity it is the teacher who most influences learning. This has been recognized by others, such as Kieran, Krainer and Shaughnessy (2013, p. 365) who stated “it is the teacher who can affect to the greatest extent the achievement of one of the main purposes of the research enterprise, that is, the improvement of students’ learning of mathematics”. In addition, in the last two decades the range of digital technology available has expanded considerably and their facilities and power have also greatly increased. In the light of these changes, the research focus of many has moved from how computers can help with learning to how teachers can make practical use of different types of digital technology to provide students with activities that will enhance their mathematical learning (Clark-Wilson, Robutti and Sinclair, 2014a; Clark-Wilson, Aldon, Cusi, Goos, Haspekian, Robutti, & Thomas, 2014b).

In addressing this new focus, it has been recognized (Thomas & Chinnappan, 2008) that there are many factors, along with some obstacles, that influence whether, and how, a teacher might use digital technology in their classroom. These include their beliefs about, and attitudes towards the technology, as well as their perception of the nature of mathematical knowledge and how it should be learned. For some teachers a positive use of digital technology requires a significant shift of mindset (Thomas, Tyrrell, & Bullock, 1996). Other researchers (e.g., Forgasz, 2006; Goos, 2005; Thomas, 2006) have identified some major obstacles to teacher use, such as a lack of time and supportive professional development, access to appropriate digital technology and poor technical support.

Simultaneously, researchers have been focusing more on the role of the teacher when using digital technology by beginning to develop theoretical frameworks that would better inform practice and research design. It is essential, as Artigue (2002) noted that “any technique, if it has to become more than a mechanically learned gesture, requires some accompanying theoretical discourse” (p. 261); where the term technique is in accordance with its definition in Chevallard’s Anthropological Theory of Didactics (ADT, Chevallard 1999). Hence, digital technological tools need to from part of the discourse as the tool can often be accompanied by its own mathematical system. For example, a calculator that embeds a computer algebra system (CAS) to find the limits of an algebraic function would offer commands that would be used in a different sequence to a “by hand” method. Hence, the challenge for teachers is to account for the mathematics of the tool as well as the mathematics that students are intended to learn to enable them to harness the epistemic value (Artigue, 2002) of the tool as an instrument. A prominent theoretical framework that has assisted in understanding this process is that of the instrumental genesis of a tool (Guin & Trouche, 1999). The framework has more recently been extended to encompass instrumental orchestration (Drijvers, Doorman, Boon, Reed & Gravemeijer, 2010; Trouche, 2004) and documentational genesis (Gueudet & Trouche, 2009), which are described more fully later in this survey paper.

In the light of the above, the purpose of this issue of ZDM is to highlight recent research that has focused on teacher use of digital technology in the learning of mathematics and how this has been accomplished. In addition, this survey paper seeks to place these articles in the context of a broad overview of some of the major new developments in this field. Our focus is on secondary school mathematics, which is explained and justified later in the paper.

The survey is structured as follows. First, we consider carefully our interpretations of the terms technology and mathematics. This foregrounds a discussion of the different and emerging ways that teaching practice employs technology to teach mathematical thinking, along with some of the challenges that this brings. Next theoretical frameworks and methodological approaches related to teacher professional learning, knowledge and practices with regard to technology use are presented, along with how these may be developed. The subsequent section looks at frameworks and studies that focus on teacher tools, resources and technologies, to include the potential role of collaboration in technology use. We conclude with some data on the impact of the coronavirus pandemic on the teaching and learning of mathematics with technology and some observations on areas that could prove fruitful for future research.

## The approach taken for the survey

Teaching mathematics with some form of digital technology is now an integral part of classroom practices in many primary, secondary and tertiary classrooms around the world. In this survey we focus primarily on school mathematics. One reason for this is that the situation at the tertiary level tends to be far more fragmented and small scale in many cases. For example, an analysis of over 300 Calculus I programs in the USA (Bressoud, Mesa & Rasmussen, 2015) reported mixed results on digital technology use, with less frequent technology use sometimes due to the high value placed on by-hand fluency by institutions. Two other related factors are the research emphasis on student learning rather than on the lecturer at the tertiary level and the types of digital technology use employed. Often this may involve lecturer demonstration, or student use may be limited to assignments in computer laboratories, both focussed on learning. Pedagogical use in lectures, such as reported by Thomas, Hong and Oates (2017), where the focus is on lecturer activity is rarer. The research on the broad impacts of technology in schools alternates between leading and lagging classroom practices. This is contrasted by two of the studies reported in this issue. Günster and Weigand’s research (2020), explores how their novel Function-Operation-Matrix theoretical framework might support the design and evaluation of tasks concerning linear functions using Geogebra technology, whereas the research of Vahey, Kim, Jackiw, Sela and Knudsen (2020) seeks to understand wider classroom practices with multi-representational technology in more naturalistic classroom settings. The literature base has been evolving since the 1980s and there are now multiple journals, edited volumes, conference proceedings and book series dedicated to the survey’s theme. Consequently, we refer to the key research activities and insights that chart the state of the art. In particular, we draw on the following previous issues of ZDM, namely:

- 41(4) Transforming Mathematics Education through the Use of Dynamic Mathematics Technologies (Hegedus & Moreno-Armella, 2009)
- 42(1) Historical aspects of the use of technology and devices in ICMEs and ICMI (Bartolini Bussi & Borba, 2010)
- 42(7) Handheld Technology in the Mathematics Classroom – Theory and Practice (Bardini, Drijvers, & Weigand, 2010)
- 49(5) Digital Curricula in Mathematics Education (Pepin, Choppin, Ruthven, & Sinclair, 2017)

This survey does not claim to be exhaustive. It draws its data from peer-reviewed sources from edited books, conference proceedings and journal articles that address our central theme of teachers and teaching with technology. Our aim is to give a broad overview of the field that provides opportunities for readers to gain deeper insights into theoretical, methodological, practical and societal challenges that concern teaching mathematics with technology.

This paper includes a summary of the contributing authors’ insights into the impact of the coronavirus global pandemic in their respective regions/countries on the teaching and learning of school mathematics in the period January—July 2020.

## Framing the study

In this section we problematize the term “technology” for the mathematics education field and position within this both the mathematics under study, and interpretations of learning and teaching in this digital context. Consequently, we reference the key research activities that frame the “state of the art” as a dialectic by offering both a historical background to the existing theoretical frames and methodological approaches and by linking these to more recent studies, to include those that feature within and beyond this Special Issue.

### What is meant by technology?

Given that the term “technology” itself has evolved to encompass multiple meanings within mathematics education, education and society at large, we begin by problematizing its definition. At a simplistic level, technology can be interpreted as the tangible ‘hardware’ devices (the computers, calculators, handhelds, mobile devices, smartphones, devices etc.) in combination with the ‘software’ or applications that offer interfaces between such hardware and users (Freiman, 2014). Indeed, in the early days of educational technology, this was often the case. However, the huge diversity of software and applications, now available in multiple formats with increasing levels of interoperability and connectivity mean that the accurate description of particular classroom contexts becomes increasingly important if we are to make sense of the field. We address this need throughout this survey paper by carefully stipulating the nature of the technology that underpinned the development of the theories and methods over the years.

On the one side, technology can be intended as a tool in the mediative sense of Vygotsky (1978), as interpreted by Bruner: “By Vygotsky's argument, tools, whether practical or symbolic, are initially "external," used outwardly on nature or in communicating with others. But tools affect their users: language, used first as a communicative tool, finally shapes the minds of those who adapt to its use.” (Bruner, 1987, p. 11). According to this interpretation, research has focused on the mediative role of technology to support students’ cognitive processes in the learning of mathematics at every stage of development. The power of semiotic mediation has been studied by Bartolini & Mariotti (2008), in which they show how: exploiting the signs involved in a mathematical task; and the system of relationships between the artefact, task and mathematical knowledge; impact on students’ learning.

On the other hand, Noss and Hoyles in their seminal 1996 text conclude, “[mathematical] representations and the communicative devices with which they are intimately bound up, can no longer be regarded as neutral players in the process of meaning making” (Noss and Hoyles, 1996, p. 41). The dual nature of mathematical tools was further developed by Hegedus and Moreno-Armella (2009) in terms of both communication infrastructures and representation infrastructures. They consider the affordances of infrastructures separately, but also as an intersection, with the products of this intersection resulting in new modes of expression (in terms of gesture, deixis and informal/formal registers). The context of their research used SimCalc MathWorlds® alongside a wirelessly networked graphic calculator system (TI-Navigator) with secondary-age students in the US. Their findings highlighted how the interplay between carefully developed tasks, which combined student physical actions with collaborative activity that culminated in shared display of their resulting graphs (in this case), impacted on the students’ learning experiences and outcomes. Although the teacher’s role was strongly alluded to, in terms of the selection of student work to display and careful posing of questions and scaffolding techniques; they concluded that further research was needed to “yield more specific pedagogical actions and related teacher knowledge around effective practice as determined by measurable learning gains” (ibid, p. 409). Indeed, this is a recurring theme in studies that concern teaching with technology in that the initial research lens is trained on the technology and the learner(s). Only when some (positive) educational impact has been established, does the research lens sometimes (but not always) move further back to bring the teacher into view within their natural setting—the classroom.

The evolution of educational technology has led the mathematics education research community to conclude that the use of digital technology has two main functions: “(a) as a support for the organisation of the teacher’s work (producing worksheets, keeping grades) and (b) as a support for new ways of doing and representing mathematics” (Sinclair & Robutti, 2020, p. 245). Towards the end of the previous century, and increasingly so in the present one, the use of technology for mathematics teachers began to have a third function: as a support for connecting, organising in communities, communicating, and sharing materials, an idea that is revisited in Sect. 6 of this survey paper. We would add a fourth function, a commercial and industry driven function, which has been to support students’ more independent work that focuses on practicing and assessing previously taught mathematical knowledge and skills in a range of online formats. Globally, such technology has been developed with limited (or no) involvement of the academic research community of mathematics educators, an issue for our field that we return to later.

A variety of taxonomies exist that seek to classify technologies more specifically with respect to their didactical function in mathematics education, such as Drijver’s, “do mathematics” and “learn mathematics” (sub divided into “practice skills” and “develop concepts”) (Drijvers 2012, p. 487). An alternative taxonomy is offered by Pierce and Stacey’s map of “pedagogical opportunities” with respect to “mathematics analysis software, such as computer algebra systems, graphics calculators, dynamic geometry and statistical packages” (Pierce and Stacey, 2010, p. 3). Their map highlights ten opportunities grouped by tasks, classroom and subject-related factors. Such taxonomies prompt researchers to define more deeply the technology under scrutiny to support the reliability and generalizability of findings, a factor that is becoming increasingly important due to the growing complexity and interoperability of emerging technologies.

### What is meant by mathematics?

The nature of the mathematics that is to be taught and learned in a classroom setting has extensive historical, cultural and political roots, which have greatly influenced the design of tools (both non-digital and digital), a topic that is addressed most comprehensively in Monaghan, Trouche and Borwein, (2016). The ZDM Issue 42(1), on the occasion of the centenary of ICME Rome 1908, examines the use of materials, artifacts and technological tools for the teaching and learning of mathematics from a historical perspective and depicts elements of the design of ‘microworlds’ as technological environments (Healy & Kynigos 2010), as well as new kinds of handheld devices for mathematical representations. The ZDM Issue 42(7) is focused on such handheld technology for teaching mathematics and examines: the new role of the teacher in managing these tools in teaching mathematics (Clark-Wilson 2010a); their position between mathematical instrument and document (Aldon 2010), to show them as representation and calculation infrastructures, available to students and teachers: the multimodality they allow in the multi-representation environment (Robutti 2010), and approaches to different mathematics topics, especially algebra, even early algebra (Zeller & Barzel 2010). Some years later, Vol. 12 of Springer MEDera Series (Calder, Larkin & Sinclair, 2018) explores not only the opportunities and constraints that mobile technologies might afford, but also the features of mobile technologies, for instance the ability to use in-built video and audio tools, which allows users to capture authentic data in their everyday world and use the data for modelling, or statistical inference.

There are inherent design decisions that are made by technology developers concerning the nature of the mathematics to be represented alongside implicit or explicit notions of a desired pedagogy (how learners are expected to learn), and the role of the teacher in the technology-mediated context. The designers of expressive technologies for the teaching and learning of mathematics have to face particular decisions on tool-mediation affordances such as dynamicity. Introduced in the mid 1980s, the dynamicity in software for geometric representations (e.g. The Geometer's Sketchpad, or Cabri-Géomètre) enabled mathematical processes such as exploration, conjecture, argumentation, and even proof (Laborde, 2000; Arzarello et al., 2002; Jackiw & Sinclair, 2009). Initially, dynamicity evolved within the context of geometry (Laborde, Kynigos, Hollebrands & Strässer, 2006), and was related to particular mathematical representations using draggable points or other objects. This dynamicity has since been conceived in a more general mathematical sense (Roschelle, Noss, Blikstein & Jackiw, 2017) and related to mathematical objects as numbers, 3D constructions, or functions, and even formulas (parameters and variables in them). It has also evolved to including more pedagogical aspects of teaching and learning by considering the dynamic acts of communicating and capturing activity over time in more (or less) effective ways, with the use of shared screens, online platforms or other resources (e.g. TI Navigator, see Robutti, 2010).

ZDM Issue 41(4), is particularly focused on dynamicity in the use of technology for approaching mathematics learning in a broader sense, to include the notions of its temporalized representation of continuous change (dynamism’s mathematical aspect), alongside the sensory immediacy of the user’s direct interaction with the mathematical representations (dynamism’s pedagogic aspect)” (Sinclair, 2009). The notion of dynamism within technological tools is founded on the vision of a mathematics that is not static, but dynamic, the mathematics of change, a topic that has extensive treatment in mathematics education research before the advent of technology. This vision was articulated by Emma Castelnuovo through the use of tools and materials (Castelnuovo, 1963), extended (still independent from technology (Schoenfeld & Kilpatrick, 2013; Kullberg, Runesson Kempe & Marton 2017) and then well-theorised in the early technological years (Kaput & Roschelle, 1998, revisited in Kaput & Roschelle, 2013). More recently, dynamism is being interpreted in multimodal environments (Noble, Nemirovsky, Wright & Tierney, 2001) to incorporate more embodied approaches. It is also evolving towards a learning perspective, supporting students in experiencing inquiry (Soldano, Luz, Arzarello & Yerushalmy, 2019), and towards a teaching perspective, supporting teachers to design authentic resources as scenarios for classroom situations (Cusi, Swidan, Faggiano & Prodromou, 2020).

The continuous evolution of technological tools deeply influences not only teaching and the professional development of teachers, but also students’ learning of mathematics. While these tools have provided the opportunity to render obsolete some kinds of traditional tasks or questions (Stacey 2002; Thomas & Holton 2003), in many countries traditional tasks and questions still prevail as part of the curriculum and its assessment (Thomas, Monaghan & Pierce 2004).

As of 2020, it is widely accepted that the use of technology per se is not sufficient to guarantee learning, an assumption that may have existed in the early days. Other areas central to discussions on learning with technology include the interaction between concepts and procedures, how new concepts, extended procedures, and structures may be approached, the thinking and reasoning that technology inspires or requires (Heid, Thomas & Zbiek, 2013) and the importance of social contexts (Pea, 1997).

A good example of this is the adoption and use of the basic calculator within primary school teaching and assessment (or not). As technologies have scaled to multiple classrooms, larger effectiveness and efficacy studies are being conducted to seek to understand not just the longer-term quantifiable outcomes of such initiatives but, equally importantly, the conditions for success. The Cornerstone Maths project in the UK, (Hoyles, Noss, Vahey, & Roschelle, 2013) and SunBay project in the US (Vahey et al, 2020) are examples of such studies. Equally important are the small-scale studies that examine individual learner impacts, with a view to designing for wider-scale use by involving teachers in the process (Sinclair, Chorney, Gunes & Bakos, 2020).

In general, there are multiple examples of computer-based applications that illustrate ways technology can enhance how children learn (different subjects) by supporting four fundamental characteristics of learning: (1) active engagement, (2) participation in groups, (3) frequent interaction and feedback, and (4) connections to real-world contexts (Roschelle, Pea, Hoadley, Gordin & Means, 2000). In particular, and focusing on mathematics, an important study situated how to use, manage and organise different technologies in the classroom, the instrumental orchestrations (Trouche, 2004), through which researchers can observe and classify these as didactic configurations (the layout of the artifacts available in the environment) with their associated exploitation modes, a concept that is addressed in depth later in this survey paper.

## Concerning classroom practice—the different and emerging place for technology

The diversity and complexity of mathematics classrooms and the learning and teaching that is designed to take place within them has resulted in an explosion of theories and research methodologies that attempt to understand their complexity. Potari writes, “the complexity lies in the classroom interactions and the ways that the teacher is balancing mathematical goals, students’ reasoning and thinking, and classroom management” (Potari, 2012, p. 1). The integration of technology undoubtedly impacts on this complexity, which we aim to illustrate in this section of the survey paper as we train our lens on the technology-enhanced classroom with an emphasis on the teacher. We begin by summarizing the research that seeks to understand and explain the nature of teachers’ classroom practices with technology—and how these practices evolve over time. We then focus on some of the identified challenges for teachers as they embed dynamic mathematical tools into their practices. We conclude the section on the teacher by considering how recognized effective teaching practices such as the real-time formative assessment of students’ learning outcomes can be reconceptualized with the use of digital tools. The students’ perspective, although not the focus of this survey paper, is touched upon given that their learning experiences (and resulting affect) within technology mediated classrooms is the main interest for teachers.

### Teachers’ classroom practices with technology and their evolution

There are few mid- to large-scale research studies in the field that have been situated in the classrooms of teachers (naturalistic studies) with the aim to both document and theorise their classroom practices with technology. This is in part a methodological issue as large-scale studies require a substantial human and financial resource to observe multiple classrooms over sufficient timescales, but also that the huge range of technological tools available to teachers would usually require specific choices of tools, rendering the findings less impactful with respect to generalizability.

The early research of Ruthven, Hennessy and Deaney in England developed approaches to support the identification and analysis of teaching expertise with technology in English secondary school mathematics and science classrooms (in particular, Ruthven & Hennessy, 2002; Ruthven, Hennessy & Deaney, 2008). Drawing on this work and a wider research literature on classroom teaching expertise, the Structuring Features of Classroom Practice (SFCP) framework was developed, containing the components: working environment, resource system, activity format, curriculum script and time economy (Ruthven, 2009). Each of these features has a set of defining characteristics and exemplifications that make it possible to devise research methods to reveal evidence from teachers’ practice within and beyond the classroom (Ruthven, 2014, p. 387). This framework has been further exemplified and validated, with increasing emphasis on particular mathematical topics within algebra and geometry (Ruthven, Deaney & Hennessy, 2009; Bozkurt & Ruthven, 2017).

An alternative theory, the Instrumental Approach applied to mathematics education (*IA*, Guin & Trouche 1999; Artigue 2002), which has its roots in cognitive ergonomics (Verillon & Rabardel 1995) and successive links to activity theory (Engeström, 1999), frames how humans (i.e. teachers and learners) become proficient users of digital tools, through the process of Instrumental Genesis (IG). The seminal studies in mathematics education took place in French upper secondary mathematics classrooms and focused on students’ instrumental genesis as they began to use CAS-enabled handheld devices (Texas Instruments’ TI-92 graphics calculators) to study functions. However, in the intervening years, the focus shifted to include teachers and the notions of Instrumental Orchestration emerged to consider the collective knowledge building of teachers and students when a technology is appropriated for some mathematical pedagogical purpose (Drijvers et al, 2010; Trouche 2004; Trouche & Drijvers, 2010). The important separation of the personal and professional aspects of teachers’ instrumental genesis is one that has been explored further by Haspekian (2005, 2014) and Clark-Wilson (2010b).

Drijvers et al. expand Trouche’s earlier work to define a typology of whole-class instrumental orchestrations (to include their didactical intentions, configurations and exploitation modes), which are classified as: Technical demo; Explain-the-screen; Link-screen-board; Discuss-the-screen; Spot-and-show and Sherpa-at work. Following a subsequent validation study, Tabach added the important Not-use-tech orchestration type to this typology (Tabach 2011). It is significant that this typology concerns the phase of classroom teaching that involves the teachers’ orchestration of whole-class work. A number of studies have adopted this framework within more holistic classroom settings, for example to include phases in the lesson when students work independently with a technology, adding Walk-and-spot (Bozkurt & Ruthven, 2016b, a) and Guide-and-explain (Simsek 2020) to the exploitation modes. These more recent studies are also seeking explore the nature of teachers’ orchestrations for the teaching of specific mathematical topics, such as geometric similarity (Simsek 2020), which reveals subtle and important differences in a more expert teacher’s practice.

In this issue Cevikbas and Kaiser (2020) describe how the implementation of a flipped classroom pedagogical approach enabled an experienced Turkish secondary school teacher to develop practices that aligned to a more socio-contructivist paradigm. This in-depth case study sheds light on how shifts in the quantity and quality of the teacher’s scaffolding and questioning techniques provided evidence of this evolution, whilst also highlighting the important affective component for the teacher involved.

#### The particular challenges of dynamic mathematical technology use in classrooms

The evolution from static (input–output) technologies to those that embed the affordances of dragging, sliders and animation to introduce a dynamic or temporal aspect to the digital environment has, since their inception, presented new challenges with respect to the role of the teacher, a result that resonates throughout the literature. The 17th ICMI Study (Hoyles and Lagrange, 2009) sought to address this theme to some extent through its two (potentially conflicting: aims: “to reflect on actual uses of technology in mathematics education, avoiding mere speculation on hypothetical prospects; and to address the range of hardware and software with a potential to impact upon or contribute to mathematics teaching and learning.” (Hoyles, Lagrange, Son & Sinclair, 2006, p. 4). However, even if the attention was on both teaching and learning, more articles focused on students’ learning than on teaching. Furthermore, the particular technologies that featured often specifically related to this, with most of the available technology used in mathematics education comprising software (generic or specific for mathematics learning), and the Web 2.0 technologies were yet to be widely used in schools.

In their review of 20 years of discourse within the technology working groups at the European Society for Research on Mathematics Education conferences, Trgalová, Clark-Wilson and Weigand comment that the design and classroom use of dynamic mathematical technologies was problematized from the very first conference (Trgalová, Clark-Wilson and Weigand, 2018). In the intervening years, classroom-based research studies have reported that, although the dynamic affordances might be present in the design of the digital environments, many teachers lacked the confidence or competence to use the dynamic aspects in their teaching (Clark-Wilson & Hoyles, 2017; Bozkurt, 2015; Simsek, 2020).

In this issue, Vahey et al (2020) qualitatively analyse videos from 24 US middle school teachers’ classroom uses of the SunBay Digital Mathematics resources to explore the nature of the teachers’ interactions with the dynamic linked visual representations contained within (Vahey et al, 2020). Situated within a large-scale professional development initiative involving 362 teachers, the project’s curricular activity system approach offered a pedagogical framework, predict-test-explain that foregrounded direct interaction with the dynamic on-screen objects. Their findings, framed within the notions of intra- and inter-representational relationships, concluded three descriptive levels of teacher use. These descriptions ranged from: no use of the technological representations by the teachers to facilitate students to report and reflect on their explorations; through to teachers’ deliberate use of the software to elicit students’ explanations based on the inter and intra-representational forms.

The notion of a dynamic view of mathematics that involves the human body has extended the theoretical field with respect to how digital representations of human movement and gesture impact on teaching and learning processes (de Freitas & Sinclair, 2013). Such embodied approaches, whilst well theorized with respect to student learning, are far less well understood with respect to wider implementations of such technologies and the perspective of the teacher. This reflects the historical pattern as new technological innovations are developed and validated within the mathematics education field. In this issue, Flood, Shvarts and Abrahamson (2020) address this gap through their notion of responsive teaching for embodied learnin*g* in which they adopt an ethnomethodology and conversation analysis approach to elicit “the ways participants take up and transform each others’ multimodal contributions to build meaning together”. The selected technology was the Mathematics Imagery Trainer for Proportions involving 23 Grade 4–6 school students from an urban school in the US state of California alongside four university mathematics education design researchers. The research concluded the following three ways in which educators can be responsive in such environments: “(1) explicitly encouraging learners to use gesture and being aware of gesture–speech mismatches; (2) using multimodal candidate understandings; and (3) co-constructing multimodally-expressed embodied ideas using gesture” (Flood et al, 2020).

#### Classroom-based assessment practices with technology

The use of digital technologies for assessment has been explored in the Springer Series *Mathematics Education in the Digital Era*, Vol. 13, (Eds. Aldon & Trgalova, 2019). In particular, the study on formative assessment with technology conducted by Cusi, Morselli and Sabena, (2019) presents design experiments of a connected classroom technology, through which students can share their work and associated reflections with both their classmates and the teacher. Such technology enables the teacher to create polls, submit them to the students, gather their answers and reveal the outcomes in real time. Cusi et al. (2019) introduce a theoretical frame to support the concept of formative assessment that considers: the functionalities of the technology (providing an interactive environment, processing and analysing, sending and displaying); the formative assessment strategies (adopting Black & William, 2009); and the agent involved (student, peer(s), teacher).

In this issue, this framework has been used by Aldon and Panero (2020) to offer a new angle on the analysis of assessment situations in a technological environment. Their contribution is twofold. Firstly, they highlight the didactic nature of the formative assessment process in relation to the mathematics at stake and how this influences the operational system in the classroom. Secondly, by considering how the use of the technology affects the structure of the classroom milieu, and the associated didactic contract, they offer examples from classrooms that provide insights into how such formative assessment strategies can be implemented from both the teacher’s and students’ perspectives.

The additional complexity that the multi-screen classroom display of students’ responses as a formative assessment approach brings to the role of the teacher was first acknowledged by Kaput and Hegedus (2002). Such situations have an impact on the development (or not) of teachers’ contingent knowledge, which is a form of tacit knowledge developed by teachers as they create and reflect on challenges that arise when technology is employed, and which may be used as opportunities to promote both teacher and student learning. This is an important research area, and one that is addressed in the next section.

#### Developing contingency—recognising and overcoming the challenges of classroom technology integration

The research literature abounds with studies that highlight how the introduction of technology to mathematics classrooms adds complexity to the role of the teacher (See Clark-Wilson, Robutti and Sinclair, 2014b). Two seminal classroom-based exploratory studies that sought to understand this complexity by Clark-Wilson (2010b) and Aldon (2011) revealed aspects of teachers’ contingent actions through their respective constructs of hiccups and didactic incidents.

Clark-Wilson’s hiccup is an epistemological construct that is defined as “the perturbation experienced by teachers during lessons stimulated by their use of the technology, which illuminates discontinuities within teachers' knowledge” (Clark-Wilson, 2010a, b p. 138). Hence, hiccups are highly contextualised to a particular teacher’s prior knowledge, experience and classroom practice with technology; alongside the particular classroom context and technology in use. The context for this study was English secondary mathematics teachers’ emergent use of the TI-Nspire handheld calculators and the TI-Navigator classroom network to privilege student explorations of variant and invariant properties. Analysis of classroom observation data revealed many examples of hiccups, alongside a set of descriptors and associated mitigating actions taken by the teachers. Both research outcomes provided evidence of teachers’ emergent contingent knowledge and practice.

Aldon’s study, which was situated in French lycée setting, also researched the use of the TI-Nspire handheld calculator, led to the definition of the didactic incident, a construct that is deeply rooted in Brousseau’s Theory of Didactical Situations and the Instrumental Approach. Broadly defined as “an event of the didactic system that modifies the dynamics of the situation” (Aldon, 2014, p. 325), a key difference in this construct and that of the hiccup is that critical incidents just occur—and may not be recognized by either the teacher or the students.

In this issue, Bozkurt and Uygan (2020) highlight the important issue of teachers’ flexibility and adaptivity as they introduce digital technologies into mathematics classrooms. By examining the case of a mathematics teacher who was a novice in technology-use, in the context of Turkish secondary school, the authors study the integration of a technology into the teaching of mathematics. The technology is a dynamic geometry software, and its integration is analysed with respect to the teacher’s professional instrumental genesis framework (Haspekian, 2014) and the hiccup construct (Clark-Wilson, 2010b). The researchers analyse the teaching practices to characterise the professional instrumentation and its evolution, with the hiccups providing “a tool for the teacher to pinpoint the differences between her professional genesis, personal genesis and pupils’ instrumental genesis” (Bozkurt & Uygam, 2020). This study extends the previous research by highlighting how the development of a teacher’s contingent knowledge was fundamental to her process of professional instrumental genesis with respect to dynamic geometry software and the role of the hiccup construct in this process.

The contingent knowledge of 11 primary school teachers in Mexico is conceptualized by the notion of pivotal teaching moments in the research of Trigueros, Sandoval and Lozano (2020), which features in this issue. “Understanding how teachers react when they are faced with an unexpected situation is important in order to gain knowledge about those particular responses that result in effective behaviors that are related to mathematics learning, and also in terms of the construction of rich learning environments that promote those kinds of behaviors.” (Sinclair et al., 2020). In Trigueros et al's study, which is contextualised within the Enciclomedia project that has created digital resources aimed at years 5 and 6, the authors examine how technology impacts the students, analysing the role of the participating teachers’ effective strategies when contingencies are used as opportunities to promote both their own and students’ learning.

## Concerning teachers’ professional learning and knowledge

In this section we survey and summarize the theoretical frameworks and methodological approaches of key studies on teachers’ knowledge concerning the use of technologies. These studies mainly consider knowledge as a product to be understood, classified and contextualised by researchers. In contrast, other studies, which may be more focused on teachers’ learning and its evolution, consider the processes teachers undergo to acquire such knowledge and competencies. Such studies collect data on the changes in teachers’ practices and corresponding justifications of such practices.

### Mathematics teachers’ knowledge concerning pedagogy and the use of technology

Several different theories exist to frame and study teachers’ knowledge and learning, which refer to different aspects of the topic. Some of them, for example, Pedagogical Technology Knowledge (PTK), and Technological Pedagogical Content Knowledge (TPACK) are specifically oriented to the knowledge, learning, and teaching of mathematics with technologies. These theories share Shulman’s (1986) common root in his research on teachers as professionals engaged in teaching. This root framed two major components of the ‘knowledge of teachers’: the content component (Content Knowledge or CK); and the pedagogical component (Pedagogical Content Knowledge or PCK), which are related respectively to the discipline and to the professional knowledge for teaching, “the particular form of content knowledge that embodies the aspects of content most germane to its teachability” (Shulman, 1986, p. 9).

In the case of mathematics, PCK concerns the intertwining of mathematics and pedagogy in relation to specific conditions of teaching the different content domains of mathematics. Ball and Bass (2003) expanded Shulman’s theoretical frame to define Mathematical Knowledge for Teaching (MKT), which they later describe as the “mathematical knowledge that teachers use in classroom to produce instruction and student growth” (Hill, Ball and Shilling, 2008, p. 374), that is the tasks in which teachers are daily engaged. If mathematics is characterised as a compression of concepts into more abstract forms, the mathematical knowledge for teaching can be seen as a sort of decompression, that aims to render them to make them explicit to learners.

**Fig. 1:**
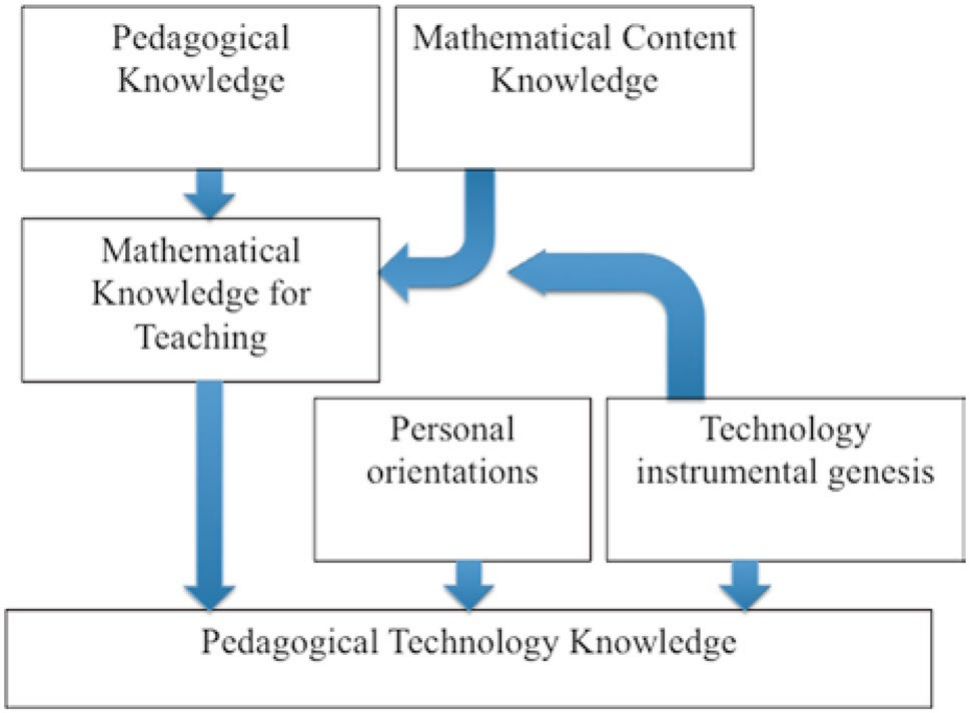
Pedagogical technology knowledge (Thomas & Palmer, 2014)

PTK and TPACK, which can be represented as graphics, both concern MKT with a focus on teachers' uses of technology. PTK (Fig. 1) combines mathematical knowledge for teaching, instrumental genesis, and personal orientation (Thomas & Hong, 2005; Thomas & Palmer, 2014) whereas *TPACK* (Mishra & Koehler, 2006) is designed to capture some of the essential qualities of knowledge required by teachers for technology integration in their teaching, whilst attending to the complex, multifaceted and situated nature of teacher knowledge.

The PTK framework is an example of an integrated set of theories as it combines MKT with (technology) IG. It also considers the important personal component of the teachers’ “personal orientations”, that is their beliefs, motivations and attitudes relating to the technology use in mathematics education. This notion formed the focus for many early studies on teacher technology integration in mathematics education as it was perceived to comprise one of the “barriers” to technology use. Bennison and Goos explored this perspective in relation to Valsiner’s (1997) zone theory, in which they reconceive the zones of free movement, proximal development and promoted action within the context of mathematics teachers’ institutional and professional contexts (Bennison & Goos, 2010).

Contrasting the two frameworks, the TPACK framework is grounded in the necessity to investigate teachers’ knowledge within the dialectic of content and pedagogy, hence its roots in Pedagogical Content Knowledge, and is expanded to incorporate the pedagogical aspects of the use of technology. Hence TPACK is a derivation of PCK with the integration of technological knowledge. By contrast PTK combines the instrumental approach with pedagogical aspects of teaching and the important consideration of teacher affect.

In this issue, Thurm and Barzel’s study used survey methods within a pseudo-experimental study in the German federal state of North Rhine-Westphalia involving 39 upper secondary mathematics teachers taking part in a professional development programme (focused on the use of multi-representational technology for an inquiry-based approach to the teaching of functions) set against a control group of 38 teachers, matched by propensity scoring, who did not. Their research revealed mixed findings with respect to the impacts of the professional development programme on the participating teachers’: (i) beliefs about teaching with technology, (ii) self-efficacy beliefs and (iii) epistemological beliefs. The most significant impact was on the teachers’ beliefs about teaching with technology (i.e. (i)), whereas there were no measured effects with respect to (ii) and (iii).

Also, in this issue, the perspective of pre-service secondary mathematics teachers in a South African context is explored in the study by Ndlovu, Ramdhany, Spangenberg and Govender (2020), which adopts the decomposed theory of planned behaviour (DTBP, Taylor & Todd, 1995) and its components (attitudes, subjective norms, and perceived behavioral control) to predict teachers’ intentions. Ndlovu and colleagues adopted a sequential mixed methods design involving questionnaire, focus group and interview protocols. Their study highlights some disparities between the student teachers’ survey and interview responses but a main finding was that their self-efficacy beliefs had the strongest influence on their control with respect to the integration of technology into their practice, accompanied by their plea for training on its use.

Ratnayake, Thomas and Kensington-Miller (2020) apply the PTK framework (called MPTK in the article in this issue, highlighting the application to mathematics) to study teachers’ improvement in the use of digital technology to design tasks for their students. Teachers’ instrumentation, their strong mathematical knowledge for teaching, including conceptual understanding, and their positive orientations are essential issues to achieve this improvement. The authors justify their use of MPTK saying that “Unlike the generic Technological Pedagogical Content Knowledge (TPACK) framework of Mishra and Koehler (2006), this [MPTK] framework focuses specifically on mathematics with its nuances of content knowledge. It incorporates the subtleties of converting DT tools into pedagogical instruments and recognises the crucial role of a teacher’s orientations in influencing their goal setting and decision making.” (Ratnayake et al, 2020).

A further article in this issue focuses on the context of pre-service teacher education in Portugal. Rocha (2020) uses three components from her own construct Knowledge for Teaching Mathematics with Technology (KTMT, Rocha 2013), to analyse the nature of the development of their knowledge as they engage in a five-stage process of task design for the teaching of functions. In the absence of actual classroom practice, the selected components (task, representations and experimentation versus justification) highlight important aspects of the knowledge and affective domains particular to a Portuguese pre-service teacher development context.

### Mathematics teachers’ learning: practices and theories

Mathematics teachers’ learning has been investigated from the point of view of communities of teachers, working together and alongside researchers/teacher educators, through the theoretical framework of Meta-Didactical Transposition (MDT) (Arzarello et al., 2014; Robutti, 2020).

MDT evolved from a 2012 study (Arzarello et al., 2012) within the Italian institutional context involving mathematics teachers engaged in professional development projects with researchers. This theory is based on the Anthropological Theory of Didactics (Chevallard & Joshua, 1985) and uses the notion of praxeology (Chevallard, 1999), which Chevallard conceived from the teacher’s perspective as a combination of practical and theoretical components, praxis and logos. The praxis concerns the practical components of a task and associated techniques to solve the task, and the logos concerns the multileveled justifications of these techniques. Praxeology in MDT extends this by also considering that of the researchers, and the theory focuses on the transposition of praxeologies from researchers (as educators) to teachers (as learners). Specifically, MDT considers the evolution of teachers’ praxeology in relation to encounters with researchers’ praxeologies, providing opportunities to trace teachers’ learning during professional development programmes over time, as evidenced by their reflections. Therefore MDT offers a framework to approach the complex study of teachers’ professional learning when researchers are introducing them to a new digital technology for the teaching of mathematics. The literature in this field has revealed that the meta-didactical praxeologies developed by teachers in their professional learning to advance in their knowledge of a technology, or to review existing ideas (see for example Taranto et al., 2020, this issue); intersect with their didactical praxeologies. This intersection can be described by the theoretical tool of MDT, called the double dialectic (Robutti, 2020).

The Italian research has expanded the framework of MDT, to enable more detailed rationales for why teachers change their praxeologies over time, such as agents that push and support such changes. In this way, praxeologies have been considered as visible macro-variables, and the agents as less visible micro-variables (Prodromou et al., 2018). In this issue, these micro- and macro-variables are integrated by Taranto, Robutti and Arzarello (2020) within the theory of connectivism, which describes the complexities of teachers’ activity within a Massive Open Online Course (MOOC) that is designed to further their use of digital technologies within mathematics teaching. The theory of connectivism frames teachers’ learning in the MOOC, as a virtual community in which each teacher’s network of knowledge can connect to the knowledge networks of colleagues. The Moodle platform adopted for the MOOC, serves as a communication and representation infrastructure that offers a virtual environment for the activation of new connections. In the article, the two theoretical frames (Meta-Didactical Transposition and connectivism) are operationalised to describe teachers’ learning about and through digital environments and resources. Hence, the teachers not only learn and work within the MOOC community, but they also implement classroom activities with their students using technologies such as GeoGebra and MathCityMap, a software that integrates a web environment (https://mathcitymap.eu) with GPS-enabled mobile devices to support maths trails (Gurjanow et al., 2017).

The processes of the design, use and evaluation of tasks for mathematical learning that involve digital technology have been increasingly investigated in recent years in relation to teachers’ professional learning. Such studies have referred to both validated approaches and related theories concerning the use of digital technology in classrooms.

The 22nd ICMI Study, on Task design included a specific theme on “Tools and representations’ (the contributions to which mostly featured digital tools) and offered the following interpretation of task, “[that which] a teacher uses to demonstrate mathematics, to pursue interactively with students, or to ask students to do something. Task can also be anything that students decide to do for themselves in a particular situation. Tasks, therefore, are the mediating tools for teaching and learning mathematics and the central issues are how tasks relate to learning, and how tasks are used pedagogically.” (Watson et al., 2013, p.10). One of the Study’s main aims was to consider how to enable mathematics teachers’ autonomous use of technological tools, with the awareness necessary at least for “(i) preparation of suitable tasks for the various age levels and many subjects appearing in the curriculum, and their introduction into textbooks, and (ii) preparing in-service teachers as well as future teachers to use them and possibly invent more of the kind” (Movshovitz-Hadar, Edri, 2013, p. 387). The resulting Study Volume synthesises the outcomes in relation to the “epistemological, mathematical, representational, and pedagogical considerations of tool-based task design” supported by examples of the theoretical frames and heuristics from the contributing authors’ research (Leung & Bolite-Frant, 2015, p. 194). Task design is also a consistent sub-theme of the European Society for Research in Mathematics Education’s 5th and 10th Topic Conferences on “Mathematics Education in the Digital Era” (Weigand, Clark-Wilson, Donevska-Todorova, Faggiano, Grønbæk, & Trgalová, 2018; Donevska-Todorova, Faggiano, Trgalová, Lavicza, Weinhandl, Clark-Wilson & Weigand, 2020).

In this issue Ratnayake et al. (2020) present a study based on Sri Lankan teachers’ involvement in digital task development during a professional development programme. The authors conclude that the increasing involvement of teachers in the design of tasks for their students forces teachers to be focused on the particular uses of digital technology in relation to their institutional constraints and epistemological choices. The effectiveness of the programme is evaluated in terms of the quality and richness of tasks produced by the teachers before and after their professional development, as evidence of improvement. As a result, the teachers design richer and more student-centred tasks compared, with respect to before. Ratnayake et al. also describe the possible reasons that underpin the teachers’ improvements, and reflect on the design and usability of tasks within secondary school teachers’ professional development programmes.

The effects of a professional development program for teaching mathematics with technology on teachers’ beliefs, self-efficacy and practices, is also addressed in this issue by Thurm & Barzel (2020 and see Sect. 5.1, for a brief description of this study).

## Digital resources and tools and their impact on teaching

This section presents the theoretical frameworks and methods that concern digital tools and resources for teaching mathematics. Starting from the Documentational Approach to Didactics, and proceeding to the more recent construct of the Resource Approach to Mathematics Education, it outlines the theoretical roots in the instrumental approach. The section concludes by expanding on the recent claim within ICMI Study 25, which focused on teachers of mathematics working and learning in collaborative groups, to consider resources and tools developed in collaborative contexts as a dialectic: the products of teachers’ collaborative work; and a means to support teachers’ collaboration.

### Resources used by mathematics teachers

The Documentational Approach to Didactics (DAD) is a theory that seeks to understand the resources used by teachers of mathematics in the broadest sense (Gueudet et al., 2012; Trouche, Gueudet & Pepin, 2020). DAD, which was introduced by Gueudet and Trouche (2009), is strongly framed in the French institutional context, and combines theoretical elements that concern:

- the use of technology (in particular the instrumental approach proposed by Rabardel and Bourmaud, 2003);
- the design of resources and curriculum (where resources are considered as having the potential to re-source in the sense of Adler (2000) teacher activity such as textbooks, digital resources, emails exchanged with colleagues, students’ activity sheets etc.);
- the teachers’ professional learning and development (not only in their work in class, but also in planning, designing, evaluating, creating assessments, discussing with parents, etc.);
- the term document to mean a given usage of a resource in a given context with a pedagogical intention.

The DAD can be applied to the study of the individual teacher, as in the research reported in this issue by Trouche, Rocha, Gueudet and Pepin (2020), to trace the documentational trajectory of a teacher’s activity over time, revealing a deeper insight into the teacher’s resource system and her associated use of resources. The use and design of digital (or non-digital) resources or teaching within such a trajectory provides data on a teacher’s development and learning over time, irrespective of whether the teacher is new to the profession or more experienced. Within Trouche and colleagues’ study, they chose a case emblematic of an experienced mathematics teacher at secondary level to reveal the trajectory concerning the digital resources, both curricular (e.g., e-textbooks, online resources) and technological (e.g., for communicating, sharing) alongside the notion of resource system as a holistic descriptor of the teacher’s activity.

Moreover, DAD is used also in the case of social situations of teachers’ use of resources, that of communities of practice (in the sense of Wenger, 1999), as teachers’ collective work in the form of networks, online associations, communities inside or outside institutional contexts, with their members’ participation, negotiation, and reification). This last aspect is quite recent and particularly important in evidencing the dialectic relationship between the development of a community of mathematics teachers, and the development of a shared repertoire of resources, also considering the social aspect of the process of learning when many people interact with many resources (Pepin & Gueudet, 2020). The Resource Approach to Mathematics Education (RAME) is an emerging research field, featuring as a volume in the series Advances in Mathematics Education (Gueudet, Pepin & Trouche, 2019). The field includes the following research domains: educational technologies and their use in the classroom; design principles for, and use of, diverse curriculum materials (especially textbooks); teachers’ professional development and the individual and collective use of its implicit resources. Fundamental to this theoretical frame is the appreciation that teachers in developed countries can be faced with many digital and non-digital resources, the careful selection of which underpins much of the craft of teaching.

The study by Bozkurt and Uygan (2020) presents a teacher’s professional learning with respect to one particular technology for teaching (i.e. Geogebra). However, there are also cases where the teacher uses multiple technologies in a class, which was first explored by Trouche (2004) or multiple teachers embrace a range of technologies (Drijvers et al, 2010), resulting in the construct of instrumental orchestration (see Sect. 4) and its further developments (Pepin & Gueudet, 2020).

The wider impact of the Instrumental Orchestration framework is explored in the article by Drijvers, Grawin and Trouche in this issue (2020). Their study adopts bibliometric clustering techniques in the field of mathematics education alongside a process of triangulation with experts to provide a sense-making sketch of the ‘landscape’ of instrumental orchestration research. The article concludes with five main descriptive clusters: Managing teaching complexity; Designing living resources; Teaching with technology; Adult learners; and Interacting with computers. Whilst acknowledging research limitations regarding the size and sampling methods for the underlying data set, the article makes a novel and interesting contribution by opening a new research strand in mathematics education through bibliometrics.

Having considered resources in relation to their wider theoretical framing, the next section considers tools as both the products of teachers’ collaboration and to support teachers’ learning in collaboration.

### The role of tools, resources and technologies in relation to teacher collaboration

Theories that frame the collaboration of teachers when working in a professional development context or within institutional settings are useful to describe the dual roles of the teacher as both a learner and a professional. The complexity of these roles is described in literature with multiple approaches, according to the particular context and theme(s) that is to be illuminated.

The ICMI Study 25 (Borko & Potari, 2020), which focused on mathematics teachers’ collaborative work, framed a resource as:

- An object that is provided by the teacher in their teaching activity of a material, socio-cultural, didactic-methodological kind. For example a lesson plan, a mathematical problem, a digital animation, etc.
- A tool that enables the retrieval and manipulation of a given resource. For example, a web browser, email, a word processor, etc.
- A tool to guide the use of a given resource. For example, a theoretical framework, a national curriculum, a school’s assessment system, etc.

The idea of a community based on the sharing of practice was first conceived in the seminal research of Wenger (1999) and it has evolved to address issues for cultivating such communities (Wenger et al., 2002), with particular applications and developments within mathematics education research.

One development addresses teachers working together as a community of inquiry, defined as any group of individuals involved in a process of empirical or conceptual inquiry into problematic situations Jaworski (2006). Jaworksi’s research explored the practices of teacher education in mathematics where inquiry implies questioning and seeking answers to problems in that context.

Another approach, within a professional development context in the region of South America, considers communities of mathematics teachers as “humans-with-media” (Borba & Villarreal, 2006), a theoretical approach supported by the belief that humans cannot be substituted by machines in teaching (Tikhomirov, 1981). This theory rejects the dualism between human and machine in favour of an inclusion of media within communities of humans. Originally developed to study mathematics teachers’ professional learning at distance, this theoretical approach is useful also for interpreting face-to-face activities, and the integration of online learning in relation to classroom activities in schools (Borba, 2012).

The advent of web 2.0 technologies expanded the opportunities for mathematics teachers’ collaborative work irrespective of whether the communities were institutional (e.g. with colleagues, trainers, researchers, policy makers etc.) or more informal (e.g. through social networks, such as Facebook or Twitter). Teachers were able to make use of emerging digital platforms, not only to upload/download materials and resources, but also to interact with each other in collaborative ways (Huang & Shimizu, 2016). Given the present pandemic era, this focus is proving useful as in every country, with or without degrees of lockdown, teachers—not only of mathematics—have been challenged to find ways to teach in online communities, sharing digital spaces and materials and leading synchronous and asynchronous learning. Alongside this, digital technologies are also providing the means for essential resources for teacher learning and support, which we address in Sect. 7.

Collaboration may be intended both within and beyond the members of particular communities if two communities (for example, one of researchers and one of teachers) work together within a professional development initiative, which may also imply negotiation, contrast, or transformation (Akkermann & Bakker, 2011). A phenomenon of boundary crossing between the two communities may occur, the underlying process for which can be managed and directed towards an equilibrium state evidenced by a crystallization of specific practices at the boundary. In this process, participants in the community usually act on a common object in a dynamic evolutive way in the sense of Star (2010), to include material and abstract forms. Applications of this theoretical construct have been recently introduced (Robutti et al., 2019) in mathematics education. In this issue Sinclair et al. (2020) apply this construct to the technological environment TouchTimes as a boundary object between two communities of researchers and teachers. The authors conceptualise the heterogeneous agencies that include boundary objects as an assemblage, a concept introduced by Deleuze and Guattari (1987) to intend the object at the boundary as a multiple dynamic entity changing over time in its components. Using this perspective, the technology TouchTimes renders possible the production of meanings on multiplication going beyond repeated addition, through the actual touching of fingers on the screen, or the rhythmic enunciation of words, or the visceral attachments to mathematical meanings. This production may arrive at a crystallization but it can also lead to disruptions, which can be useful in themselves to help—in this case—primary teachers in being confident in the use of a technology for producing meanings on multiplication, and the consequent adoption of it, or integration of it into their teaching practice. This interpretation is consistent with that introduced in Robutti, Aldon, Cusi, Olsher, Panero, Cooper, … & Prodromou (2019) to describe the complex structure of boundary objects, comprising different components but offering a new perspective, which is the interactions within communities that highlight the fragility of collaboration, with its provisional and mutable nature, made visible through such disruptions.

One group of the ICMI Study 25 was focused on tools and resources in collaborative groups of teachers working and learning together. This group is producing an analysis of the tools and resources both for teachers’ collaboration and resulting from teachers’ collaboration (Robutti, Trouche, Cusi, Psycharis, Kumar & Pynes, forthcoming).

## Early implications of the coronavirus global pandemic on the teaching and learning of mathematics with technology

The editors of this special issue took the opportunity to gather data on the impacts of the coronavirus pandemic on the teaching and learning of mathematics given that, in many countries the use of technology became a vital tool for maintaining some continuity for students’ education.

### Methods

The contributing authors were surveyed, which provided data on the following countries (and/or specific geographical regions: Canada (Alberta and British Colombia), France, Germany, Italy, Mexico, Netherlands, Turkey, United Kingdom (England) and the United States.

- We sought to understand the context at the start of the pandemic in spring 2020, by asking:
- Did primary and secondary schools close, and if so, did national or regional expectations with respect to teaching and assessing mathematics change as a result?
- How prepared were primary and secondary school teachers to respond to the changed expectations and what professional support was offered?
- Was any research initiated that sought to understand the impacts of the pandemic on teaching and learning mathematics, with a particular emphasis on the technological tools adopted?

### Findings

All respondents reported that both the primary and secondary schools closed and that in all countries teachers were responsible for continuing to teach mathematics to students at home. The national examinations for both primary and secondary age students were cancelled in Canada (Alberta and British Colombia) and England, replaced by processes of teacher assessment. In Germany, there were differences according the federal state. In some cases the national examinations were postponed, in other states, they were cancelled. In Mexico, there were difference between the public and private schools. The national examinations for primary and secondary public schools were postponed. In Turkey, the grade 8 national examinations went ahead as a paper and pencil environment in the school with students wearing face masks and respecting social distancing policies. Respondents reported an increased emphasis on teacher assessments and the use of existing school data to determine these.

The survey participants’ perceptions of the teaching workforce’s preparedness to teach mathematics ‘online’ is summarised in Table 1.

**Table 1:** A summary of teachers’ preparedness to teach mathematics online

	Teachers were unprepared to teach mathematics ‘online’				Teachers were highly prepared to teach mathematics ‘online’
	
Primary teachers	Canada (Ontario and British Columbia) France Germany Netherlands Mexico UK (England) USA	Germany (Bavaria)	Italy Turkey		
Secondary teachers	France Netherlands Mexico	Canada Germany UK (England) USA	Turkey	Italy	

The national or regional provision of guidance to teachers on how to teach mathematics in this context differed greatly between countries. In some countries, there was a government led response on a national scale. For example, in France, the Centre National d’Enseignement à Distance (CNED, National Center for Distance Education, https://www.cned.fr/) was funded by the Ministry of Education to create a platform Ma Class à la Maison (My class at home, https://www.cned.fr/maclassealamaison). On this platform the teachers of students of all ages (primary, college and lycee) could create a "virtual class" and choose and assign their own content or select from existing contention the platform. The students were automatically subscribed to the virtual class. In the UK (England) the government funded the development of an online school (Oak National Academy, https://www.thenational.academy/), which offers video-rich online mathematics lessons for primary and secondary age learners, although a recent mobile phone survey of teachers in England (n = 6058) revealed that, in the state-funded sector, only 3% of primary teachers and 2% of secondary teachers planned to align their school curriculum with that of Oak National Academy in the 2020/21 school year (Teacher Tapp, 20 July 2020). In Turkey, the Ministry of National Education (MNE) had begun in 2012 to design an online teaching platform Eğitim Bilişim Ağı (EBA, Educational Information Network). Following the school closures, the EBA was developed immediately by MNE and mathematics teachers were enabled to access and offer mathematical content to their students that included lesson videos, summaries of the units, exercises and tests. Teachers had opportunities to share any of these files to all of the selected students at any time although some videos were broadcast on an EBA TV channel as some students did not have access to the internet. In other countries, teachers were expected to select their own content and overcome the challenges to distribute this content either digitally or physically to students. In Mexico, teachers and students exchanged activity sheets by taking and sending photos on mobile phones or via home visits.

As of August 2020, there are limited research findings with respect to the impacts of Covid 19 on the teaching of mathematics with technology. One collaborative research study has been initiated that is contrasting teachers’ perspectives in Netherlands, Belgium (Flanders) and Germany revealing aspects of the teachers’ and students’ affect, experiences and competences to use digital technologies during the pandemic. Emerging findings suggest that teachers’ didactic approaches for distance lessons varied greatly between the three geographies with respect to the following constructs: rehearsing and practicing; introducing new topics; conceptual understanding; and procedures and algorithms. The results reveal differences that might be explained by ministerial guidelines, local access to particular technologies and teachers’ confidence to use online synchronous tools such as video conferencing (Drijvers, 2020).

In England, with fieldwork in schools undesirable but an urgent need to learn from schools' responses to physical closures, Nesta (www.nesta.org) partnered with SchoolDash (www.schooldash.com) to undertake a data analysis of EdTech usage, supported by the UK Department for Education. By collaborating directly with the creators of four popular online mathematics teaching platforms, platform data was collected and analysed for usage patterns before and during lockdown (running from Monday 24 February 2020 to Sunday May 30). The research sampled data from all schools (primary and secondary, state and independent) and explored not only at the amount by which usage of these services increased, but also at the shifting patterns with respect to school type and location, levels of student engagement, and the types of devices used (Nesta, 2020). The study placed a particular emphasis on the nature of the digital divide between poorer students and their more affluent peers. The findings highlighted that “usage during lockdown increased considerably and whilst, before lockdown, it was common for these products to be used disproportionately by schools with fewer poor pupils, located in more affluent areas or with higher Ofsted [statutory school inspection] ratings. To some degree, these imbalances were reduced, or even reversed, during lockdown. However, these previously under-represented schools often showed lower levels of student engagement during lockdown. As a result, gaps in student activity (as opposed to teacher activity) frequently widened. Furthermore, students at these relatively disadvantaged schools were more likely to access online learning platforms using phones rather than computers.” (Hannay, 2020).

These early studies highlight themes that are yet to be fully understood in the global context of technology use in mathematics education, such as the digital divide, relationships with large technology companies and the ethical implications for conducting research that reaches into students’ homes.

## Future research areas

First, we consider what the survey reported in Sects. 2 to 6 suggests in terms of the potential for future research in general involving teacher use of technology in secondary mathematics. Second, and most importantly, as we write this survey paper in the midst of a global pandemic, we ask what kind of research might be needed to understand teaching mathematics with technology in a mid- and post-pandemic world where mathematical teaching and learning is situated in different environments. For example, in the absence of (real) classroom observation protocols, there are both ethical and practical challenges to overcome for researchers to be able to “observe” synchronous and asynchronous teaching and learning, particularly for younger children or situations with poor digital access.

Two major related areas would benefit from closer study. The first is to examine teacher professional development that will assist them to integrate technology into their classrooms. The second is research that looks at the outcomes of longer-term use of technology in teachers’ own mathematics classrooms. One of the common threads here is that teachers have often commented to us that the professional development they have attended has lacked relevance to their classroom experience. What they ask for are resources that are practical and easily adapted for their use. There are several approaches that could be considered here. One that has historically been used is to provide teachers with such resources. However, another that appears to have positive effects is to work with groups of teachers who form supportive communities in which to develop practical resources that they feel comfortable to use in their school environment. Once these are developed researchers can then support teachers as they implement the ideas in their classrooms at the same time employing frameworks, such as that of Ruthven (2009) or Chevallard’s ATD, to research factors and practices or praxeologies that are perceived to lead to positive implementation and student learning.

Another area where further investigation could ascertain possible benefits to the learning of mathematics would be how to harness the value of increasing connectivity of digital technology devices. One example, seen in Thomas, Hong and Oates (2017) is where the teacher used student smartphones to good effect. In this case the students and teacher employed KakaoTalk on a Social Network Service to send and receive messages and pictures via the screen of a smartphone, enabling immediate pedagogical feedback during an undergraduate class. The potential of such teacher-led interactions and what they demand of the teacher in terms of technology use and instrumental orchestrations could be a very fruitful field of research.

Measuring effects of a digital technology intervention on student learning is not always easy. In fact behavioural, cognitive and emotional engagement may be used as a predictive proxy for learning (Kahu & Nelson, 2018). Furthermore, investigating what links exist between effective teacher practice with digital technology in the classroom and gains in learning is an area that would benefit from further consideration in research (Hegedus & Moreno-Armella, 2009).

Another potential area for consideration, where we still don’t know the answer in spite of past research, is whether and to what extent digital technology may change the mathematics that students learn. Part of the complexity here is that the epistemic value of digital instruments is intimately bound up with the teacher perspective of mathematics and of the role of technology in learning. For example, Heid, Thomas and Zbiek (2013) asked how prolonged experience with computer algebra systems would affect how students understand and use algebraic symbols, and we could add, what is the potential role of the teacher in this? This is closely related to the issue of student construction of technology-related schemes and what teacher practices in terms of classroom presentation and discussion of techniques might enable these.

Finally, the mathematics education research community should and cannot ignore the extensive development in educational technology (EdTech) that is being driven by an estimated $2.6 billion global industry (HolonIQ, 2019), much of which has been developed with limited reference to existing research in mathematics education and/or close involvement with the educational research community. Groundbreaking projects such as EDUCATE in London have been designed to bridge this gap for early stage EdTech enterprises by offering educational research methods training and mentoring using approaches that align with product and business development goals (Cukurova, Luckin & Clark-Wilson, 2019). However, with educational technology products that include mathematics content now reaching millions of users worldwide, there is a need for closer involvement of the mathematics education research community to support companies to adopt more evidence-led approaches both in the design and evaluation of their products. This might be achieved by the community becoming more enterprising and seeking to raise funds to develop and commercialise its own innovations or by entering into collaborative research or co-design partnerships with companies that aim to formatively evaluate the effectiveness of existing products. The Erasmus-funded European EdTech Network aims to broker opportunities for Higher Education Institutions in Europe (and beyond) to connect with the EdTech industry to share research knowledge and collaboration experiences through its information portal and events (www.eetn.eu).

Globally, it is the large EdTech companies that are generating big data sets that are ripe for the development of learning analytics, dashboards and artificially intelligent algorithms that enhance or personalize learners’ and teachers’ experiences for a range of purposes. However, traditional research designs to evaluate the educational effectiveness for such systems become problematic when learners’ and experiences within such systems become “unique”, as are the experiences of their teachers and lecturers. Furthermore, the ethical implications of such automations are yet to be fully understood whereby a particular learner’s mathematical diet might be restricted or enhanced in ways that promote educational inequities or bias.
